# ARID1A deficient undifferentiated spindle cell and rhabdoid sarcoma of the prostate: report of a unique case with emphasis on diagnostic implications

**DOI:** 10.1186/s13000-022-01198-4

**Published:** 2022-02-06

**Authors:** Wenjuan Xu, Haiying Dong, Guoqing Ru, Ming Zhao

**Affiliations:** 1 Cancer Center, Department of Pathology, Zhejiang Provincial People’s Hospital, Affiliated People’s Hospital, Hangzhou Medical College, Hangzhou, 310014 Zhejiang China; 2 Urology & Nephrology Center, Department of Urology, Zhejiang Provincial People’s Hospital, Affiliated People’s Hospital, Hangzhou Medical College, Hangzhou, 310014 Zhejiang China

**Keywords:** Prostate, Stromal sarcoma, *ARID1A*, Biallelic inactivation, Case report

## Abstract

**Background:**

SWItch Sucrose Non-Fermentable (SWI/SNF) chromatin-remodeling complex functions collectively as a tumor suppressor and the inactivation of any of its constituent components is frequently associated with tumor initiation and/or progression. Most SWI/SNF deficient tumors share common rhabdoid morphology. ARID1A is the most frequently dysregulated SWI/SNF subunit in human cancer and inactivation of ARID1A is frequent across carcinomatous types while very rarely drives the tumorigenesis of sarcomas. Herein, we report a rare case of primary prostatic undifferentiated spindle cell sarcoma with focal rhabdoid morphology, harboring biallelic inactivation of *ARID1A* detected by next-generation sequencing with complete loss of ARID1A expression by immunohistochemistry.

**Case presentation:**

The patient is a 58-year-old man who presented with dysuria and obstructive voiding symptoms for 3 month and was found to have a large, ill-defined, prostatic mass lesion with circumferential extension into the rectal wall on imaging studies. A needle biopsy showed a spindle cell undifferentiated sarcoma of the prostate and the patient was treated by chemotherapy of combined etoposide and cisplatin for 2 months. A subsequent imaging study showed that the tumor was significantly enlarged, and the patient underwent laparoscopically radical prostatectomy. Gross examination showed a disrupted, 10 × 7 × 5 cm, solid and cystic mass involving almost the entire prostate and sparing the seminal vesicle glands. Histologic examination showed that tumor was composed mainly of mildly atypical, oval to spindle-shaped cells, arranged in sheets and fascicles or herringbone-like patterns within a small amount of edematous to myxoid, vascularized stroma. Notably, groups of discohesive rhabdoid tumor cells with eccentric nuclei, prominent nucleoli, and abundant globular cytoplasm were observed. There were prominent mitotic figures, multifocal geographic necroses, and foci of lymphovascular invasion. Immunohistochemistry showed that the tumor cells were diffusely positive for TLE-1 and vimentin and focally positive for epithelial membrane antigen, AE1/3, Cam5.2, SATB2, and CD34 (all in less than 10% tumor cells). Next-generation sequencing showed biallelic inactivation mutation of *ARID1A*; the predicted inactivating effect of ARID1A deletion was confirmed by immunohistochemical staining. After the surgery, the patient received an alternative combined chemotherapy of doxorubicin and ifosfamide for 5 months. The patient died 9 months after initial presentation due to extensive abdominal metastases.

**Conclusions:**

We report an ARID1A deficient undifferentiated spindle cell and rhabdoid sarcoma of the prostate, adding to the growing spectrum of SWI/SNF driven undifferentiated sarcoma. Rhabdoid cells can be a helpful morphological clue for promoting molecular and immunohistochemical analyses for deficiency of SWI/SNF subunits, in the diagnostic workup of undifferentiated neoplasms featuring epithelioid or rhabdoid morphology.

**Supplementary Information:**

The online version contains supplementary material available at 10.1186/s13000-022-01198-4.

## Background

The SWItch Sucrose Non-Fermentable (SWI/SNF) chromatin-remodeling complex is a large multi-subunit protein assembly that orchestrates chromatin compaction and accessibility for gene transcription in an ATP-dependent manner [1–3]. The mammalian SWI/SNF chromatin-remodeling complex consists of 15 subunits encoded by 29 genes, some of which are recurrently mutated in human cancers including *ARID1A*, *ARID1B*, *ARID2*, *PBRM1*, *SMARCB1*, *SMARCA4*, and others [1–3]. From a simplified oncopathogenic point of view, the SWI/SNF chromatin-remodeling complex functions collectively as a tumor suppressor [1–3]. Accordingly, the inactivation of any of its constituent components is frequently associated with tumor initiation and/or progression [1–4]. In soft tissue neoplasms, SMARCB1 (INI1) is the subunit most frequently inactivated, followed by SMARCA4 (BRG1), and SMARCA2 [5, 6]. Inactivation of ARID1A is frequent across carcinomatous types and very rarely drives the tumorigenesis of sarcomas [7, 8]. Herein, we report a rare case of primary prostatic undifferentiated spindle cell sarcoma with focal rhabdoid morphology, harboring biallelic inactivation of *ARID1A* detected by next-generation sequencing (NGS) with complete loss of ARID1A expression by immunohistochemistry (IHC).

## Case presentation

**Fig. 1:**
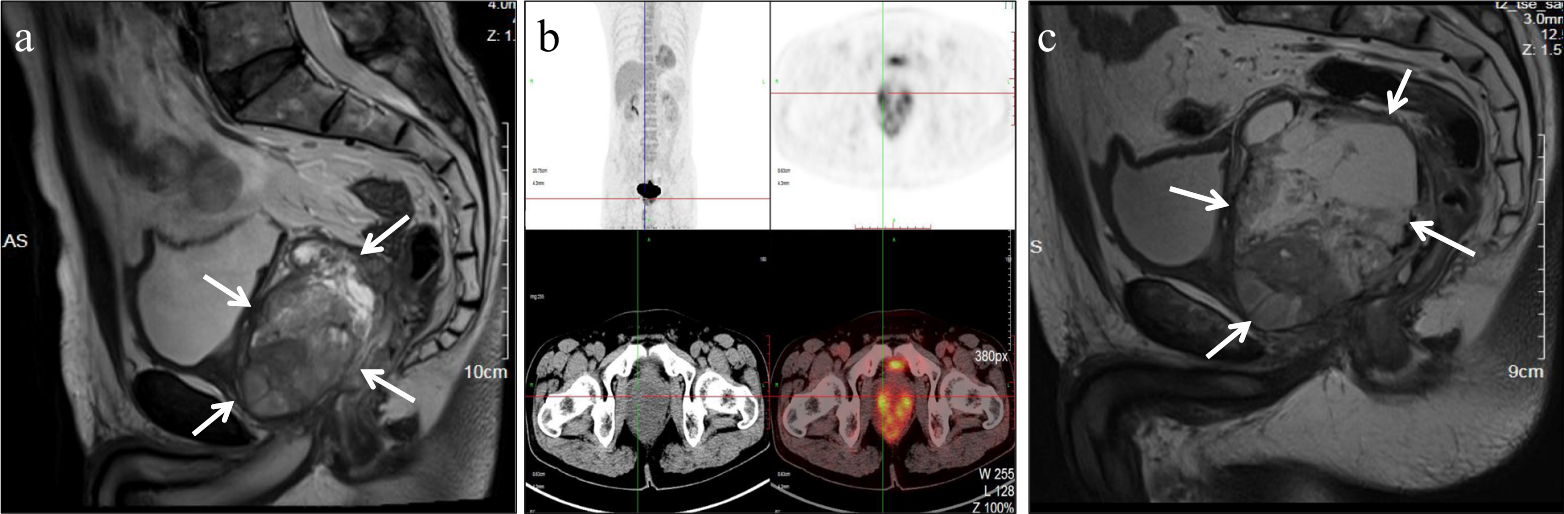
Imaging features of ARID1A deficient undifferentiated spindle cell and rhabdoid sarcoma of the prostate. (**a**) Magnetic resonance imaging (MRI) revealing a large, ill-defined, prostatic mass lesion with circumferential extension into the rectal wall (*as indicated by arrows*). (**b**) Positron emission tomography-computed tomography scan showing a mixed mass of the prostate with an uneven increase in fluorodeoxyglucose metabolism. (**c**) MRI showing that the prostatic tumor is significantly enlarged with central areas of hemorrhage, necrosis and cystic change (*as indicated by arrows*), after 2 months of chemotherapy with etoposide and cisplatin

**Fig. 2:**
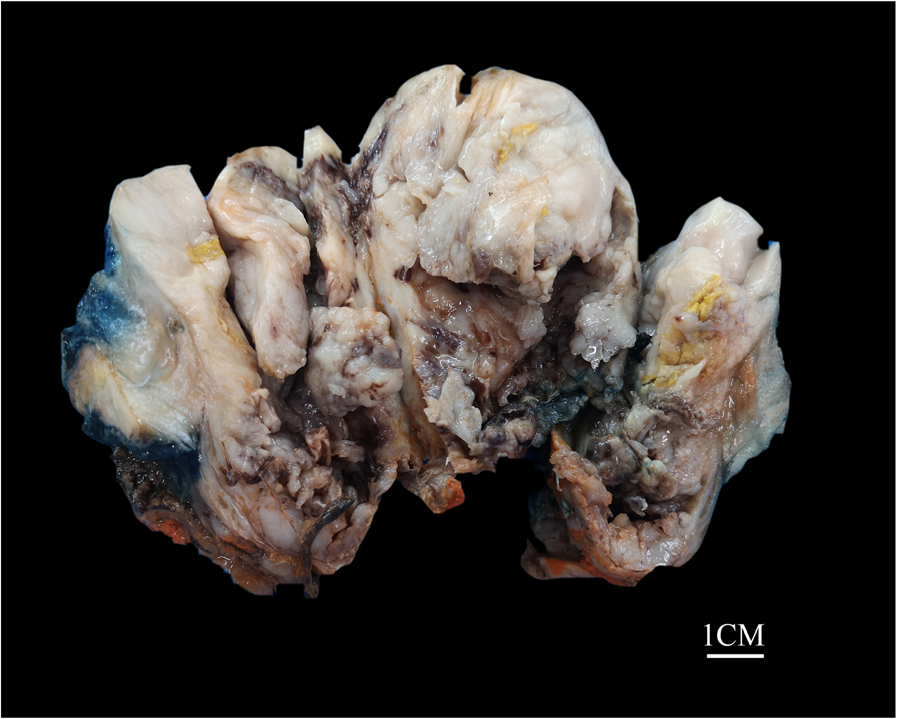
Macroscopic features of ARID1A deficient undifferentiated spindle cell and rhabdoid sarcoma of the prostate. Gross examination showing a disrupted, 10 × 7 × 5 cm, solid and cystic mass involving almost the entire prostate

**Fig. 3:**
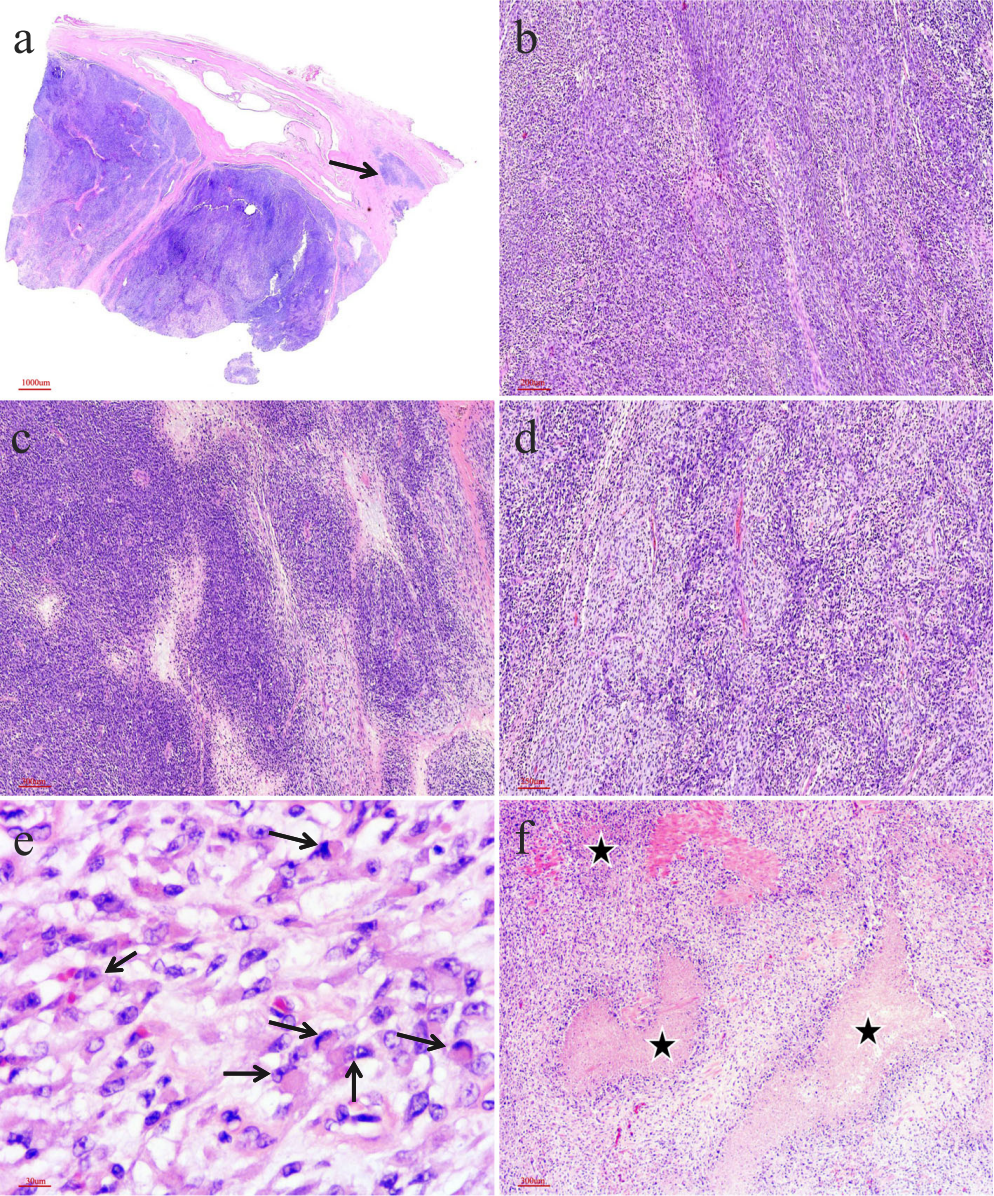
Histologic characteristics of ARID1A deficient undifferentiated spindle cell and rhabdoid sarcoma of the prostate. (**a**) Lower magnification showing a densely cellular, solid and lobulated tumor with an overall pushed but multifocal infiltrating border (*as indicated by arrow*). (**b, c**) The tumor cells were mainly mildly atypical, oval to spindle-shaped cells, arranging in sheets and fascicles or herringbone-like patterns within edematous to myxoid, vascularized stroma. (**d**) Areas of storiform, whirling or morular structures are noted. (**e**) Groups of discohesive rhabdoid tumor cells (*as indicated by arrows*) and (**f**) multifocal geographic necroses (*as indicated by asterisks*) are evident

A previously health 58-year-old man with an unremarkable medical history presented with dysuria and obstructive voiding symptoms for 3 month. Digital rectal examination revealed a firm to hard, fixed, pelvic mass compressing the anterior aspect of the anal canal. By laboratory examination, the serum carcino-embryonic antigen (2.3μg/l) and prostate-specific antigen (PSA, 3μg/l) were within normal limits. Magnetic resonance imaging (MRI) revealed a large, ill-defined, prostatic mass lesion with circumferential extension into the rectal wall (Fig. 1a). Positron emission tomography-computed tomography (PET-CT) scan showed a mixed mass of the prostate, with an uneven increase in fluorodeoxyglucose metabolism, considering a malignant tumor (Fig. 1b). There was no evidence of local or distant metastasis by imaging studies. A needle biopsy of the prostatic mass was performed which showed a slightly pleomorphic spindle cell sarcomatoid tumor with brisk mitoses and multiple tumor necroses, without any specific lineage differentiation by extensive IHC investigations. A diagnosis of spindle cell undifferentiated sarcoma was rendered and the patient was treated by multi-agent chemotherapy combination of etoposide and cisplatin for 2 months. However, a subsequent enhanced MRI showed that the tumor was significantly enlarged with central areas of hemorrhage, necrosis and cystic change (Fig. 1c). Multiple enlarged bilateral inguinal lymph nodes were also identified. The patient underwent laparoscopically radical prostatectomy with bilateral pelvic lymphadenectomy. Although the mass was densely adherent to the rectum wall, complete gross surgical resection was achieved. Macroscopic examination of the prostatectomy specimen showed a disrupted, 10 × 7 × 5 cm, solid and cystic mass with a fresh, grey white to tan cut-surface and multiple foci of necrosis, involving almost the entire prostate and sparing the seminal vesicle glands (Fig. 2). Histologically, lower magnification showed a densely cellular, solid and lobulated tumor, with an overall pushed but multifocal infiltrating border, affecting most part of the prostatic parenchyma (Fig. 3a). The tumor was composed predominantly of mildly atypical, oval to spindle-shaped cells containing scant eosinophilic cytoplasm and oval to elongated nuclei with small distinct nucleoli, arranged in sheets and fascicles or herringbone-like patterns within a small amount of edematous to myxoid, vascularized stroma (Fig. 3b, c). Minor areas of storiform, whirling or morular structures were also noted (Fig. 3d). Notably, groups of discohesive rhabdoid tumor cells with eccentric nuclei, prominent nucleoli, and abundant globular cytoplasm were observed (Fig. 3e). There were prominent mitotic figures (up to 10/10 high power fields), multifocal geographic necroses (Fig. 3f), and foci of lymphovascular invasion. Scattered in the tumor, there were entrapped prostatic ductal structures which occasionally showed basal cell hyperplasia and squamous metaplasia. Eleven pelvic lymph nodes were free of tumor.

**Fig. 4:**
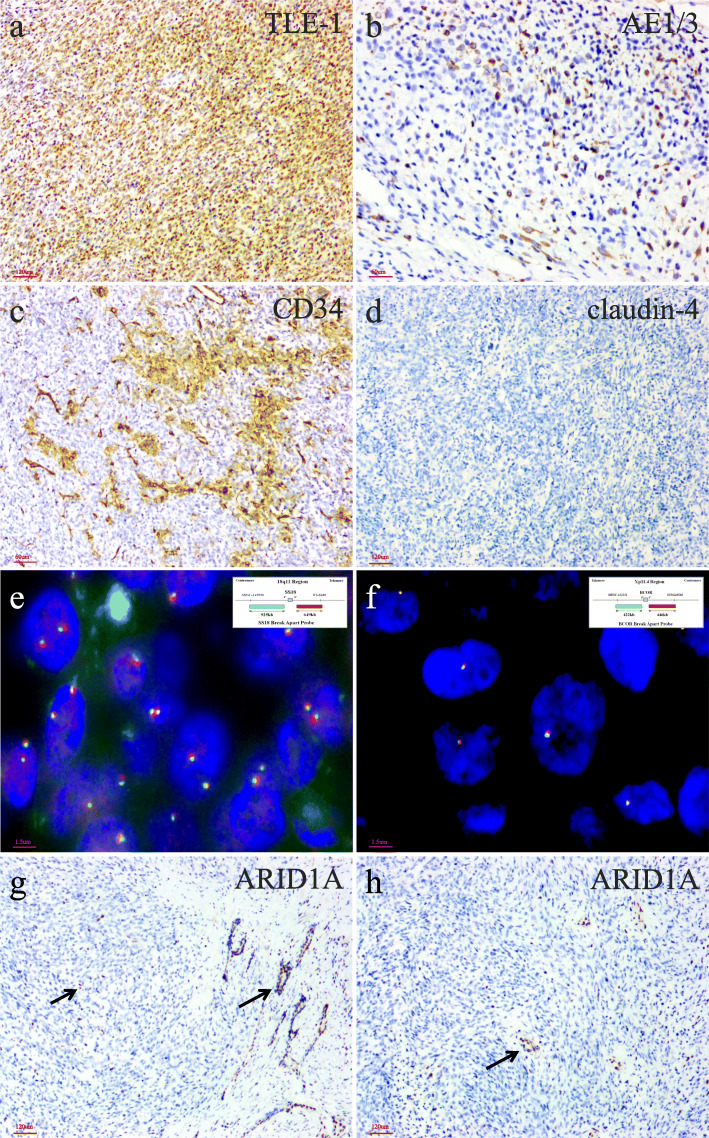
Immunohistochemical and molecular genetic analyses of ARID1A deficient undifferentiated spindle cell and rhabdoid sarcoma of the prostate. The tumor cells are diffusely positive for (**a**) TLE-1 and focally positive for (**b**) AE1/3 and (**c**) CD34, and are negative for (**d**) claudin-4. Fluorescence in-situ hybridization analyses revealing negative for rearrangements of both the (**e**) *SYT/SS18* and (**f**) *BCOR* loci (*Insets* in **e** and **f** indicating schematic diagram of break-apart probes flanking *SYT/SS18* and *BCOR*, respectively). (**g, h**) ARID1A immunohistochemistry exhibiting that tumor cells show complete loss of nuclear ARID1A expression with retained strong reactivity in the native prostatic glands and background stromal cells (*as indicated by arrows*)

By IHC, the tumor cells were diffusely positive for TLE-1 (Fig. 4a) and vimentin and focally positive for epithelial membrane antigen, AE1/3 (Fig. 4b), Cam5.2, SATB2, and CD34 (Fig. 4c) (all in less than 10% tumor cells). Immunohistochemical stains for SOX10, S100 protein, P63, cytokeratin5/6, NKX3.1, PSA, alpha-methylacyl-CoA racemase (AMACR), progesterone receptor, STAT6, CD117, DOG1, cyclinD1, smooth muscle actin, desmin, myogenin, HMB45, and claudin-4 (Fig. 4d) were all negative in the tumor cells. The expression of SMARCB1/INI-1, SMARCA4/BRG-1, and H3K27me3 was retained and the Ki67 proliferation index was estimated at 50% (Additional file 1). With the suspicion of a primary synovial sarcoma or a *BCOR* rearranged sarcoma, fluorescence *in-situ* hybridization (FISH) analyses were performed which revealed negative for rearrangements of both the *SYT/SS18* and *BCOR* loci (18q11.2, *SS18/SYT* Break Apart Probe Kit; Xp11.4, *BCOR* Break Apart Probe Kit; Anbiping, China) (Fig. 4e, f)*.* A representative formalin-fixed, paraffin-embedded block of tumor tissue was selected for somatic mutation analysis by NGS on FoundationOne CDx panel, a hybrid-capture panel that targets 576 cancer-relevant genes. The NGS results were listed in Table 1. Pathologic mutations were identified in frameshift deletions of ARID1A exon5 p.Leu657Glnfs*18 (c.1970_1971del) and ARID1A exon18 p.Thr1535Argfs *36 (c.4604_4605del)*.* IHC using anti-ARID1A antibody (clone D2A8U, Roche) showed complete loss of expression on the tumor cells (Fig. 4g, h). Based upon the combination of morphologic, immunohistochemical, and molecular features, a diagnosis of ARID1A deficient undifferentiated spindle cell and rhabdoid sarcoma of the prostate was rendered. After the surgery, the patient received an alternative combined chemotherapy of doxorubicin and ifosfamide for 5 months. Thereafter, PET-CT scan revealed multiple tumor metastases in the bilateral obturator muscles, sigmoid colon, and rectum. The patient died 9 months after initial presentation.

**Table 1:** Genetic alterations identified by next-generation sequencing

Gene	Transcript Number	Nucleotide Change	Amino Acid Alteration	Exon Position	Coding Effect	Variant Allele Frequency
Pathogenic findings						
*ARID1A*	NM_006015.5	c.1970_1971 del	p.Leu657Glnfs *18	Exon 5	Frameshift	45.08%
*ARID1A*	NM_006015.5	c.4604_4605 del	p.Thr1535Argfs *36	Exon 18	Frameshift	42.79%
Variants of uncertain significance						
*ATM*	NM_000051.3	c.7886_7890 del	p.Ile2629Serfs *25	Exon 53	Frameshift	39.95%
*STK11*	NM_000455.4	c.328_329 del	p.Val110Hisfs *52	Exon 2	Frameshift	40.44%
*FLCN*	NM_144997.6	c.1691_1692 del	p.His564Profs *95	Exon 14	Frameshift	15.81%

## Discussion

Deleterious mutations of genes encoding subunits of the SWI/SNF complex are commonly found in more than 20% of all human cancers [1–4]. Such mutations are thought to promote tumorigenesis by disturbing transcriptional homeostasis due to impairment of DNA repair and chromatin remodeling in transcribed genes [1–4]. *ARID1A* is located in 1p36.11 and encodes a key noncatalytic member of the SWI/SNF chromatin-remodeling complex. The encoded protein ARID1A, also known as BAF250a or SMARCF1, is the most frequently dysregulated SWI/SNF subunit in human cancer [7]. Mutations in *ARID1A* are frequent across carcinomatous types, present in over 30–50% of ovarian and endometrial carcinomas, 10% of hepatocellular and urinary bladder carcinomas, and 5–10% of colorectal, gastric, and non-small cell lung carcinomas [7, 9]. ARID1A functions primarily as a tumor suppressor in most of these tumor types; in the absence of ARID1A, defects in control of enhancer activity impair developmental programs and cause extensive dysregulation of gene expression, thus driving tumor formation [9]. However, data of *ARID1A* mutations in soft tissue neoplasms are limited and evidence suggest that *ARID1A* mutations are very rare in sarcomas. In a recent comprehensive genomic profiling assay of 584 soft-tissue sarcomas of various histologic variants, only 12 cases (2%) showed SWI/SNF subunits alterations including inactivating mutations or genomic deletions, of which the most common mutated subunit was ARID1A (4; 0.7%), followed by PBRM1 (3; 0.5%), SMARCB1 (3; 0.5%), and ARID2 (2; 0.3%) [8]. *ARID1A* inactivation has also been found in pediatric neuroblastoma and aggressive meningioma [7, 9, 10]. For the current case, the *ARID1A* p.Leu657Glnfs*18 detected in the patient’s tumor is a frameshift mutation, which is located in exon 5 of the *ARID1A* and causes the encoded protein sequence to undergo continuous erroneous changes starting with leucine at position 657 until position 18 is replaced with a stop codon. The detected *ARID1A* p.Thr1535Argfs*36 is also a frameshift mutation that is located in exon 18 of the *ARID1A*, causing the encoded protein sequence to undergo continuous erroneous changes starting with threonine at position 1535 until position 36 is replaced with a stop codon. The biallelic inactivation mutation of *ARID1A* may lead to premature termination of protein coding, resulting in truncated protein products, which may affect the function of the protein. As *ARID1A* mutations are believed to result in reduced gene expression [9], we performed immunohistochemical staining with a specific antibody against ARID1A, which revealed complete loss of expression in all tumor cells, further confirming the loss-of-function mutation of *ARID1A* in this prostatic tumor. From a morphologic point of view, SWI/SNF deficient soft tissue neoplasms usually consist of monomorphic undifferentiated epithelioid to plump spindle cells with some of rhabdoid cytomorphology [5, 6]. The prototypical example that illustrates such morphologic changes is malignant rhabdoid tumor, which is caused by *SMARCB1* biallelic inactivation in virtually all cases [11]. Given its roles in coordinating cellular differentiation and proliferation, inactivation of the SWI/SNF complex often results in an undifferentiated cellular state, which may be responsible for the universal non-pleomorphic undifferentiated cytomorphology in most SWI/SNF deficient neoplasms [4, 5]. These findings suggest that the identified ARID1A deletion, which is the alternative pathway of SWI/SNF inactivation, may be involved in the rhabdoid morphology seen in the current case. Given the monomorphic undifferentiated spindle cells with focal rhabdoid phenotype and loss of ARID1A expression by IHC, we believe that biallelic inactivation mutation of *ARID1A* discovered by NGS represents the driven molecular event in the current case.

Primary prostatic sarcoma is an exceedingly rare type of non-epithelial malignant tumor accounting for less than 0.2% of all prostate cancers [12–14]. Typically, these tumors demonstrate high-grade morphology with pleomorphic spindle to epithelioid cells, brisk mitoses and necroses, and the disease course is rapid and portends a short and dismal prognosis. Most prostatic sarcomas are diagnosed on adult patients and among which leiomyosarcoma represents the most commonly seen histologic type, followed by rhabdomyosarcoma (RMS), and prostatic stromal sarcoma [12–14]. However, almost all histologic types of bone and soft tissue sarcomas can occur primarily in the prostate. Prostatic stromal sarcoma is traditionally considered as a diagnostic category that groups malignant mesenchymal neoplasms of the prostate that are not classifiable as more specific tumor types [15, 16]. However, a recent NGS study has suggested that prostatic stroma sarcoma is a cytogenetically heterogeneous neoplasm in which a subset of cases can be further reclassified as specific entities on the basis of identification of specific gene alternations [16]. In that study, using next-generation DNA and RNA sequencing, the authors identified pathogenic molecular abnormalities in 19 of 22 cases of primary prostatic mesenchymal neoplasms of possible specialized prostatic stromal origin, including cases originally diagnosed as prostatic stroma sarcoma (11 cases) and stromal tumor of uncertain malignant potential (11 cases) [16]. Recurrent genetic variants included *TP53* alterations in 6 cases, *ATRX* mutations in 2 cases, and gene rearrangements in 8 cases. Among the latter, 4 gene fusions identified (*NAB2-STAT6, JAZF1-SUZ12, TPM3-NTRK1,* and *BCOR-MAML3*) can be used for reclassification of the cases as specific entities. Among the 9 cases that lacked *TP53* mutations, *ATRX* mutations and oncogenic gene fusions, similar to our case, one high-grade unclassified prostatic sarcoma was described to harbor a pathogenic biallelic inactivation of *ARID1A.* This case was a 19-years-old male patient whose tumor showed a solid, short fascicles or patternless pattern with necrosis and increased mitosis [16]. In addition, an adult embryonic RMS of the prostate reported recently by Olivas and Antic [17] had point mutation of *ARID1A* plus mutations in *KRAS*, *PIK3CA*, and *EP300*. These data suggest that *ARID1A* mutations may not be non-recurrent genetic events in high-grade prostatic sarcomas. For those with specific differentiation, such as rhabdomyoblastic differentiation, it is unclear whether the detected mutations of *ARID1A* represent oncogenic drivers or just passenger mutations. Accumulation of more cases along with functional studies of ARID1A inactivation are needed for further elaboration.

Despite extensive immunohistochemical stains along with multiple molecular analyses, the lineage differentiation of the current case is difficult to elucidate and the differential diagnostic spectrum is board. Diffuse and strong expression of TLE-1 as wells as focal expression of multiple epithelial markers strongly suggest the diagnosis of synovial sarcoma, and rhabdoid differentiation can also be seen in synovial sarcoma [18]. However, synovial sarcoma usually does not express CD34, and harbors the genetic hallmark of *SYT/SS18* rearrangements. Undifferentiated spindle cell tumor background with focal rhabdoid cells makes embryonic RMS and malignant peripheral nerve sheath tumor with rhabdomyoblastic differentiation (malignant triton tumor) also enter into the differential diagnosis. However, negative for myogenic markers including desmin and myogenin, can readily exclude the possibility of embryonic RMS, and absence of expression of S100 protein and SOX10 together with retained expression of H3K27me3 disagree with a malignant triton tumor [19]. Perhaps the most important differential diagnosis is sarcomatoid carcinoma of the prostate, and *ARID1A* mutations have rarely been documented in prostate carcinomas [9]. Sarcomatoid carcinoma of the prostate is usually admixed with recognizable prostatic adenocarcinoma but rarely maybe monophasic, and most cases are associated with prior radiotherapy or hormone therapy that represent dedifferentiation from (usually high grade acinar) prostatic adenocarcinoma. In this context, detailed clinicopathologic correlation and careful evaluation of multiple sections for the presence of an underlying prostate carcinoma are important [20]. In addition, studies have suggested that staining for the epithelial tight junction protein claudin-4 may be useful, as SWI/SNF deficient soft tissue neoplasms in general lacked claudin-4 expression, whereas more than 80% of SWI/SNF deficient undifferentiated carcinomas exhibited expression of this protein [21, 22]. Absence of claudin-4 immunoreactivity for our case further supports its mesenchymal neoplasm nature. Taken together, we believe the current case does not fit any of the well-defined prostatic sarcoma categories other than undifferentiated sarcoma as a diagnosis of exclusion.

In summary, we describe a rare case of primary prostatic sarcoma that shows undifferentiated spindle cell with focal rhabdoid morphology and harbors biallelic inactivation of *ARID1A* detected by NGS with complete loss of ARID1A expression by IHC. Our case adds to the growing spectrum of SWI/SNF driven undifferentiated sarcoma. Because rhabdoid morphology is characteristic of SWI/SNF deficient neoplasms, its recognition can be helpful in the diagnostic workup of undifferentiated neoplasms featuring epithelioid or rhabdoid morphology, in combination with molecular and immunohistochemical analyses for deficiency of SWI/SNF subunits. Inclusion of other SWI/SNF immunomarkers, such as ARID1A, in the differential diagnostic workup of poorly differentiated sarcoma not fitting well-known subtypes, such as *SMARCB1* and *SMARCA4*-deficient sarcomas, is recommended. As ARID1A loss has been associated with improved response to immunotherapy across diverse tumor types [23] and ARID1A can directly interact with EZH2 to antagonize EZH2-mediated interferon response [24], identification of *ARID1A* mutations in aggressive soft tissue sarcomas may of great clinical significance for targeted therapy.

## Supplementary Information

**Additional file 1.** Additional pertinent immunohistochemical features of ARID1A deficient undifferentiated spindle cell and rhabdoid sarcoma of the prostate. The tumor cells are diffusely positive for (A) vimentin and focally positive for (B) epithelial membrane antigen, (C) Cam5.2, and (D) SATB2 (all in less than 10% tumor cells). (E) The Ki67 proliferation index was estimated at 50%. Immunohistochemical stains are negative in the tumor cells including (F) SOX10, (G) S100 protein, (H) P63, (I) cytokeratin5/6, (J) NKX3.1, (K) alpha-methylacyl-CoA racemase (AMACR), (L) smooth muscle actin, (M) desmin, (N) myogenin, (O) DOG1, (P) STAT6, and (Q) CD117. The expression of (R) H3K27me3, (S) SMARCB1/INI-1, and (T) SMARCA4/BRG-1is retained.

## Data Availability

Records and data pertaining to the case are in the patient’s secure medical records in Zhejiang Provincial People’s Hospital, Affiliated People’s Hospital, Hangzhou Medical College. All searched data by literature review are included in this paper.

## Abbreviations

AMACR: alpha-methylacyl-CoA racemase
FISH: Fluorescence *in-situ* hybridization
IHC: Immunohistochemistry
MRI: Magnetic resonance imaging
NGS: Next-generation sequencing
PET-CT: Positron emission tomography-computed tomography
PSA: Prostate-specific antigen
RMS: Rhabdomyosarcoma
SWI/SNF: SWItch Sucrose Non-Fermentable

## References

[1] Kadoch C, Hargreaves DC, Hodges C, Elias L, Ho L, Ranish J, Crabtree GR (2013). Proteomic and bioinformatic analysis of mammalian SWI/SNF complexes identifies extensive roles in human malignancy. Nat Genet.

[2] Masliah-Planchon J, Bièche I, Guinebretière JM, Bourdeaut F, Delattre O (2015). SWI/SNF chromatin remodeling and human malignancies. Annu Rev Pathol.

[3] Wang X, Haswell JR, Roberts CW (2014). Molecular pathways: SWI/SNF (BAF) complexes are frequently mutated in cancer--mechanisms and potential therapeutic insights. Clin Cancer Res.

[4] McBride MJ, Kadoch C (2018). Disruption of mammalian SWI/SNF and polycomb complexes in human sarcomas: mechanisms and therapeutic opportunities. J Pathol.

[5] Agaimy A (2019). SWI/SNF complex-deficient soft tissue neoplasms: a pattern-based approach to diagnosis and differential diagnosis. Surg Pathol Clin.

[6] Schaefer IM, Hornick JL (2021). SWI/SNF complex-deficient soft tissue neoplasms: an update. Semin Diagn Pathol.

[7] Mathur R (2018). ARID1A loss in cancer: towards a mechanistic understanding. Pharmacol Ther.

[8] Lucchesi C, Khalifa E, Laizet Y (2018). Targetable alterations in adult patients with soft-tissue sarcomas: insights for personalized therapy. JAMA Oncol.

[9] Wu RC, Wang TL, Shih IM (2014). The emerging roles of ARID1A in tumor suppression. Cancer Biol Ther.

[10] Abedalthagafi MS, Bi WL, Merrill PH, Gibson WJ, Rose MF, du Z, Francis JM, du R, Dunn IF, Ligon AH, Beroukhim R, Santagata S (2015). ARID1A and TERT promoter mutations in dedifferentiated meningioma. Cancer Genet.

[11] Hollmann TJ, Hornick JL (2011). INI1-deficient tumors: diagnostic features and molecular genetics. Am J Surg Pathol.

[12] De Bari B, Stish B, Ball MW (2017). Adult prostatic sarcoma: a contemporary multicenter rare Cancer network study. Prostate.

[13] Wang X, Liu L, Tang H, Rao Z, Zhan W, Li X, Zeng H, Zhang P, Wei B, Lin T, Wei Q, Lu Y, Li X (2013). Twenty-five cases of adult prostate sarcoma treated at a high-volume institution from 1989 to 2009. Urology.

[14] Musser JE, Assel M, Mashni JW, Sjoberg DD, Russo P (2014). Adult prostate sarcoma: the memorial Sloan Kettering experience. Urology.

[15] Herawi M, Epstein JI (2006). Specialized stromal tumors of the prostate: a clinicopathologic study of 50 cases. Am J Surg Pathol.

[16] Acosta AM, Sholl LM, Dickson BC, McKenney JK, Gordetsky JB, Pins MR, Marino-Enriquez A, Dong F, Dubuc AM, Cin PD, Fletcher CDM (2021). Re-evaluating tumors of purported specialized prostatic stromal origin reveals molecular heterogeneity, including non-recurring gene fusions characteristic of uterine and soft tissue sarcoma subtypes. Mod Pathol.

[17] Olivas AD, Antic T (2020). Rhabdomyosarcoma of the adult prostate: a case report with complete molecular profile. Int J Surg Pathol.

[18] Jun SY, Choi J, Kang GH, Park SH, Ayala AG, Ro JY (2004). Synovial sarcoma of the kidney with rhabdoid features: report of three cases. Am J Surg Pathol.

[19] Ito Y, Kohashi K, Endo M, Yoshimoto M, Ishihara S, Toda Y, Susuki Y, Kawaguchi K, Furukawa H, Tateishi Y, Yamada Y, Kinoshita I, Mori T, Yamamoto H, Nakashima Y, Oda Y (2021). Clinicopathological and prognostic significance of H3K27 methylation status in malignant peripheral nerve sheath tumor: correlation with skeletal muscle differentiation. Virchows Arch.

[20] Hansel DE, Epstein JI (2006). Sarcomatoid carcinoma of the prostate: a study of 42 cases. Am J Surg Pathol.

[21] Schaefer IM, Agaimy A, Fletcher CD (2017). Claudin-4 expression distinguishes SWI/SNF complex-deficient undifferentiated carcinomas from sarcomas. Mod Pathol.

[22] Rekhtman N, Montecalvo J, Chang JC, Alex D, Ptashkin RN, Ai N, Sauter JL, Kezlarian B, Jungbluth A, Desmeules P, Beras A, Bishop JA, Plodkowski AJ, Gounder MM, Schoenfeld AJ, Namakydoust A, Li BT, Rudin CM, Riely GJ, Jones DR, Ladanyi M, Travis WD (2020). SMARCA4-deficient thoracic Sarcomatoid tumors represent primarily smoking-related undifferentiated carcinomas rather than primary thoracic sarcomas. J Thorac Oncol.

[23] Okamura R, Kato S, Lee S, Jimenez RE, Sicklick JK, Kurzrock R (2020). *ARID1A* alterations function as a biomarker for longer progression-free survival after anti-PD-1/PD-L1 immunotherapy. J Immunother Cancer.

[24] Bitler BG, Aird KM, Garipov A, Li H, Amatangelo M, Kossenkov AV, Schultz DC, Liu Q, Shih IM, Conejo-Garcia JR, Speicher DW, Zhang R (2015). Synthetic lethality by targeting EZH2 methyltransferase activity in ARID1A-mutated cancers. Nat Med.

