# Supplementary figures and images for: ARID1A deficient undifferentiated spindle cell and rhabdoid sarcoma of the prostate: report of a unique case with emphasis on diagnostic implications
Source: Diagn Pathol. 2022 Feb 6;17:22. doi: 10.1186/s13000-022-01198-4 (PMC8818209; doi:10.1186/s13000-022-01198-4)

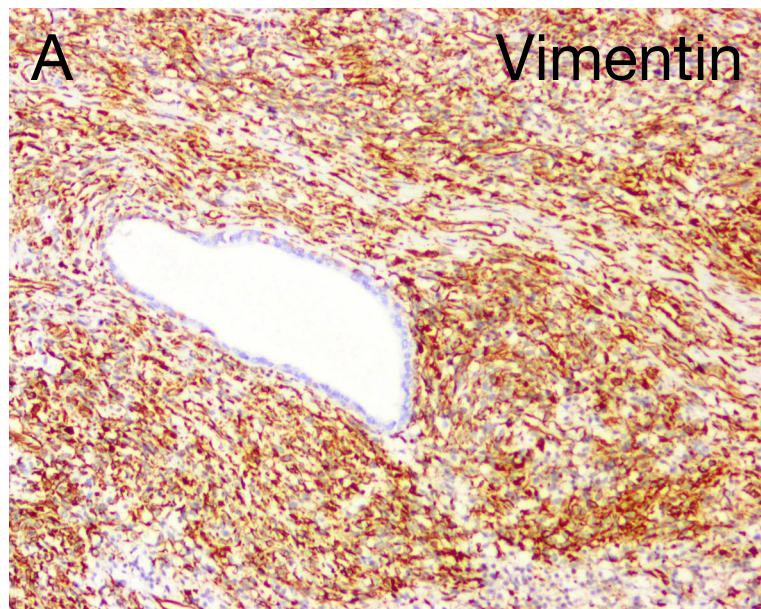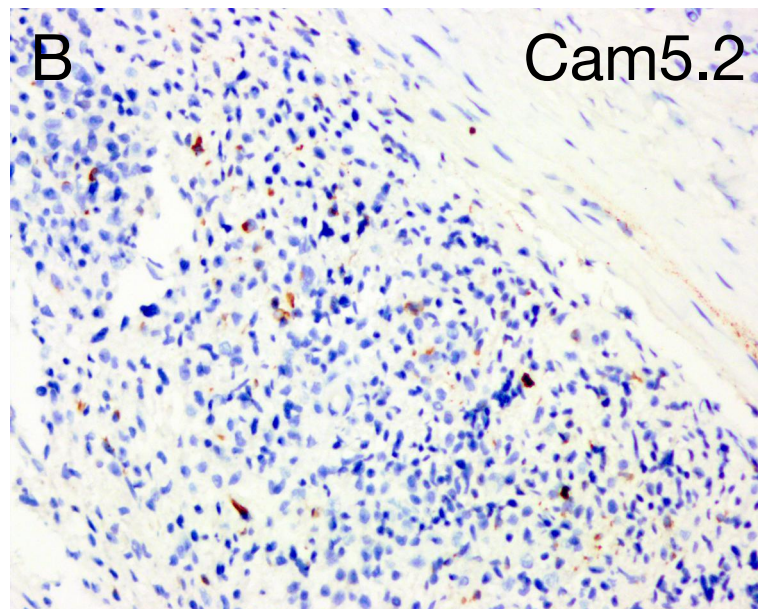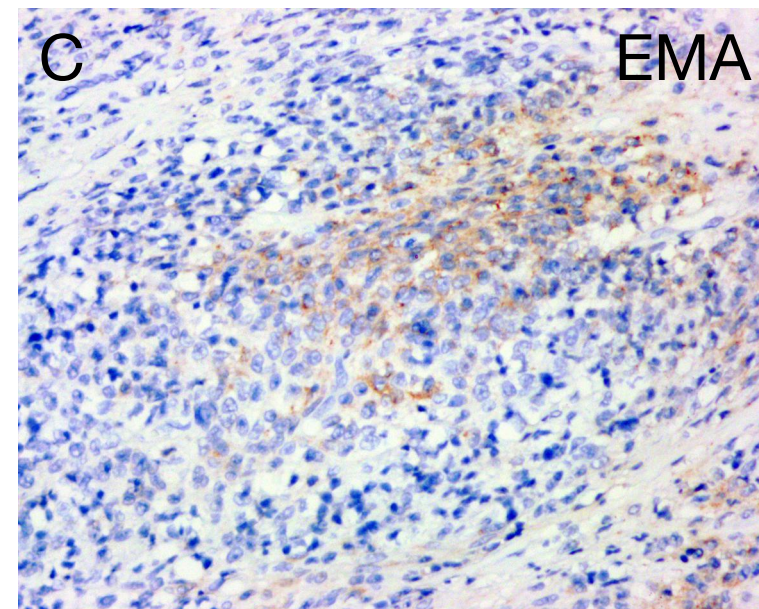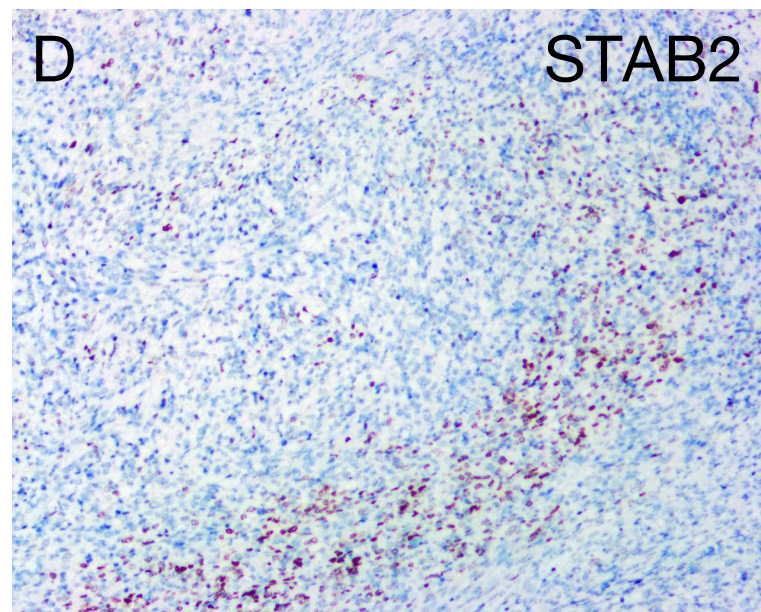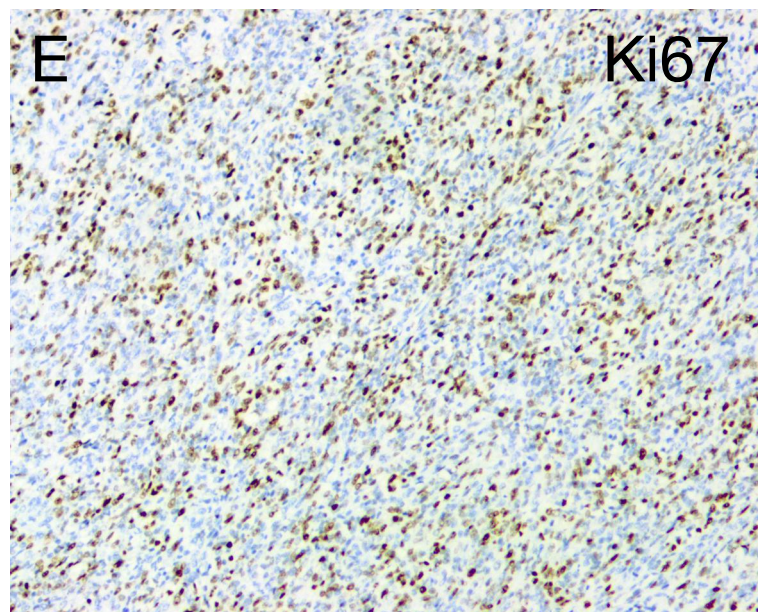

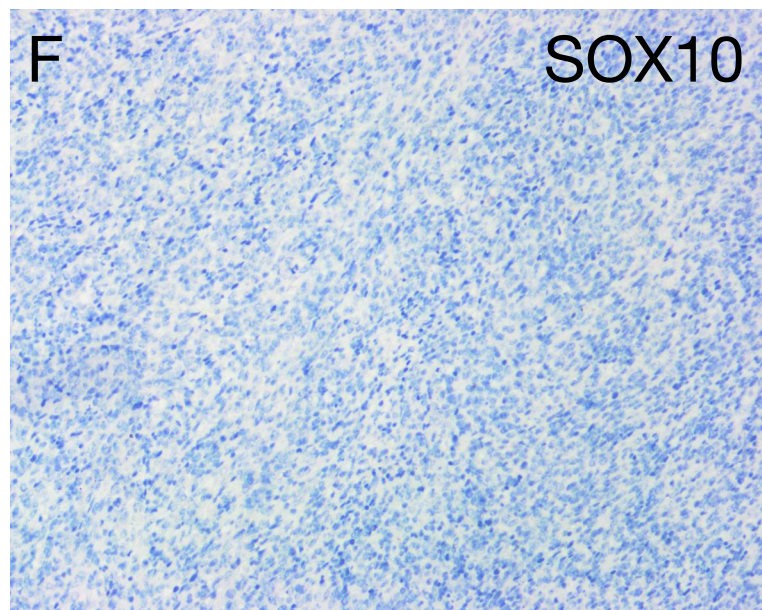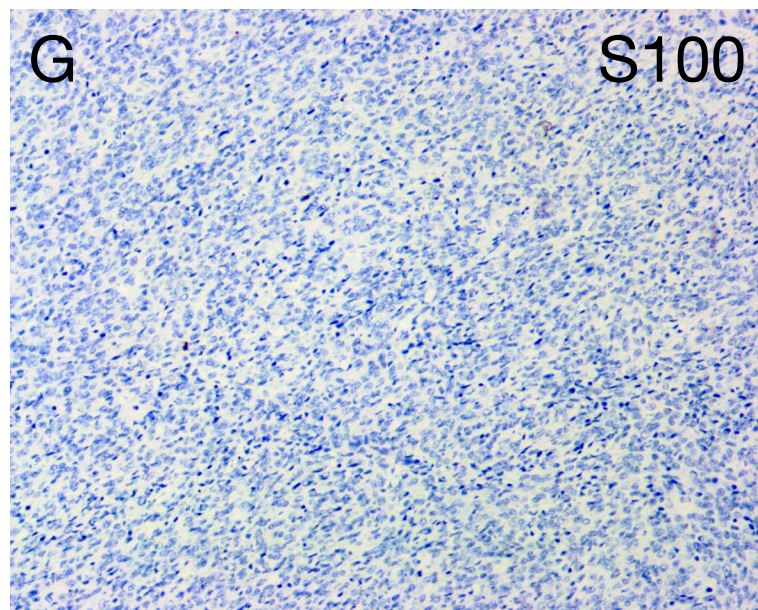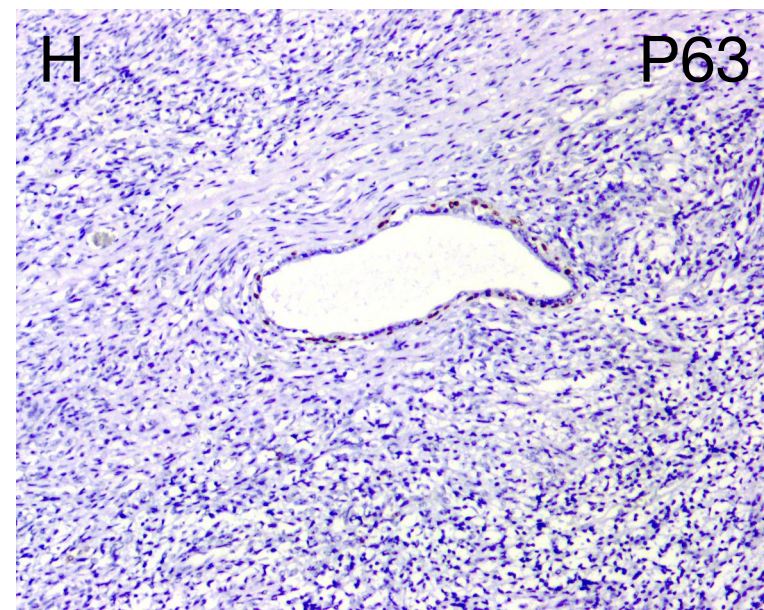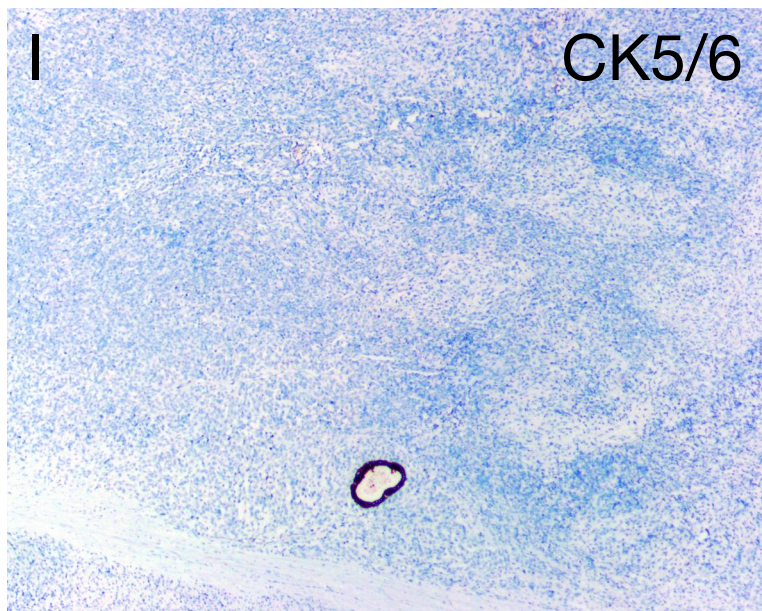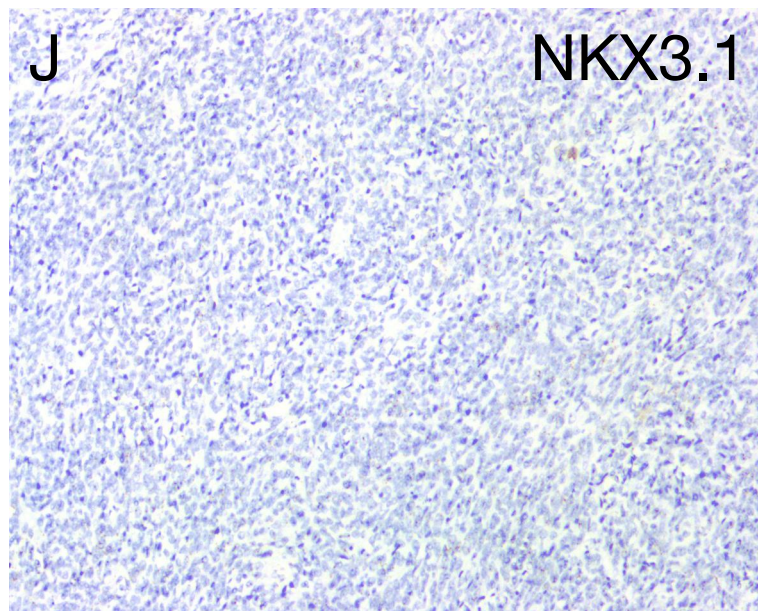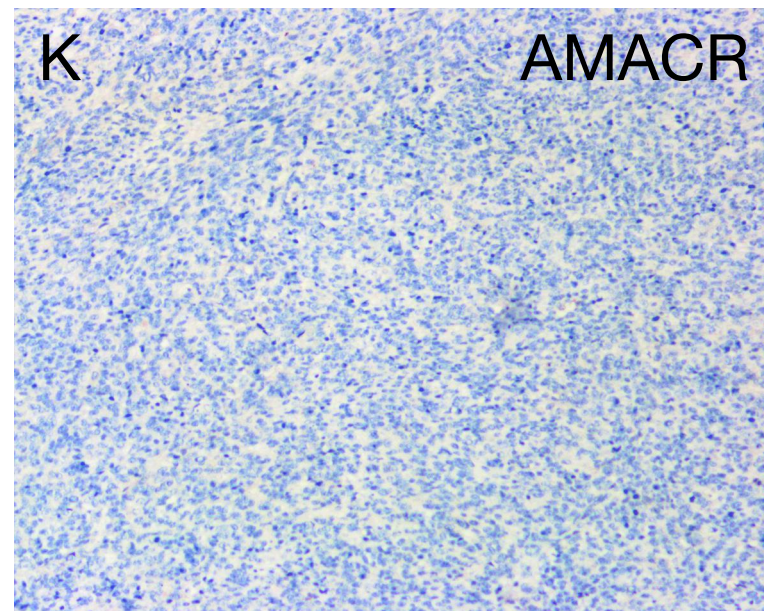

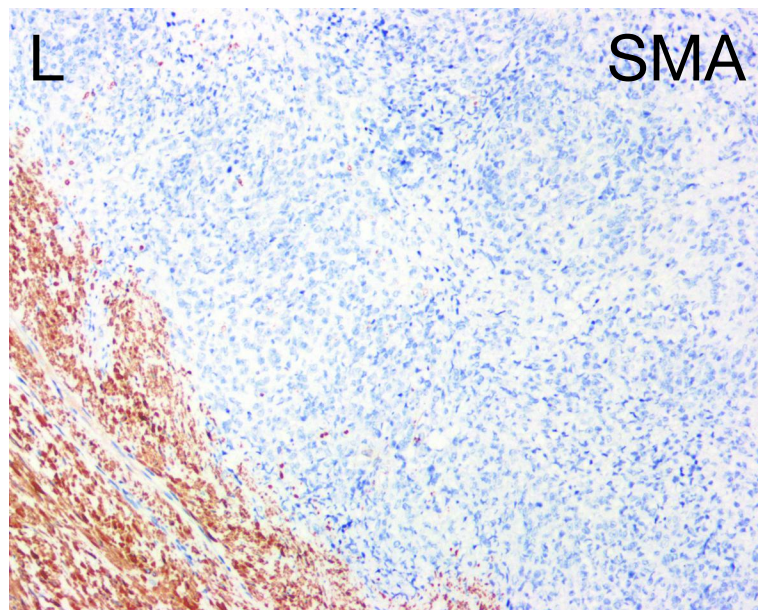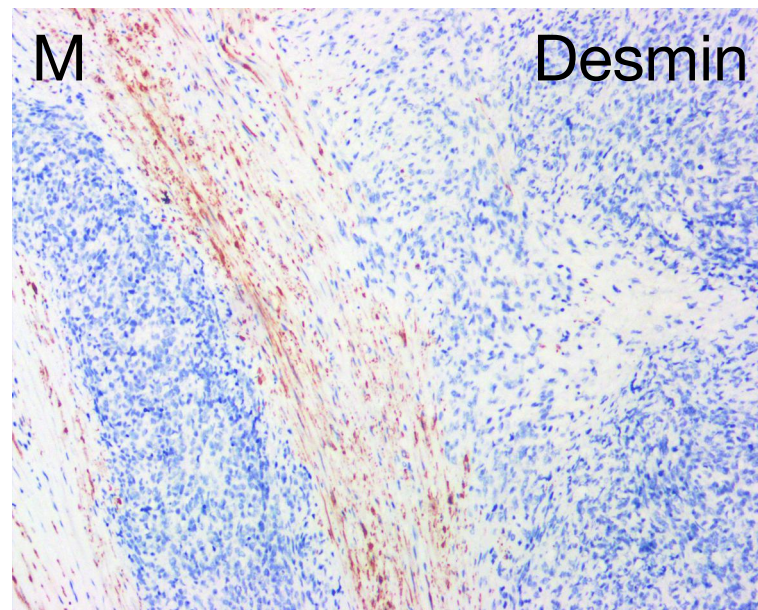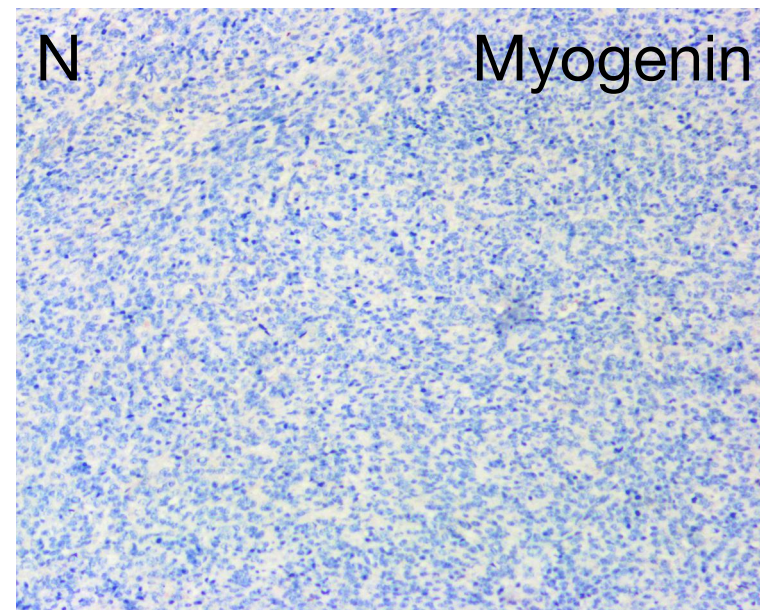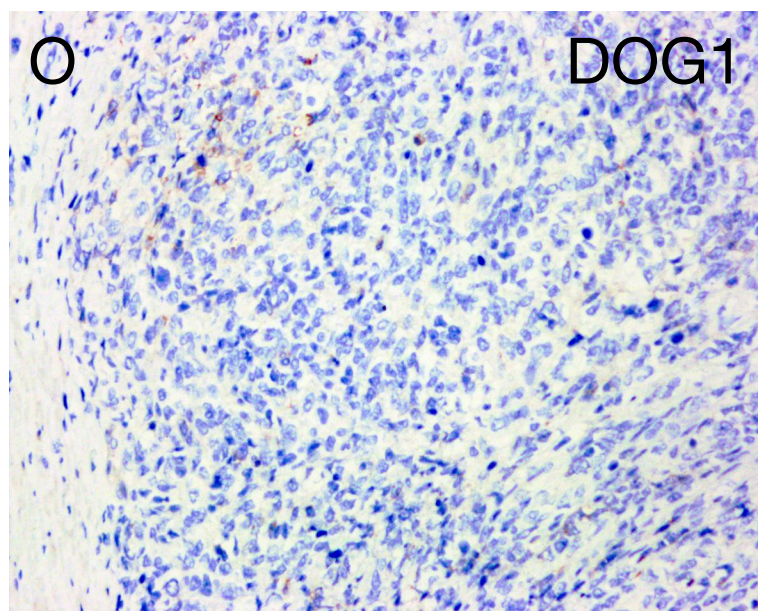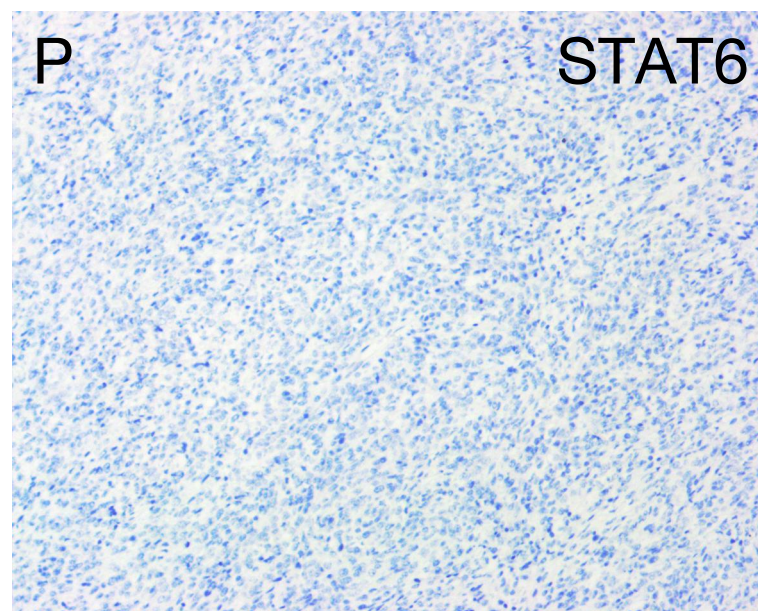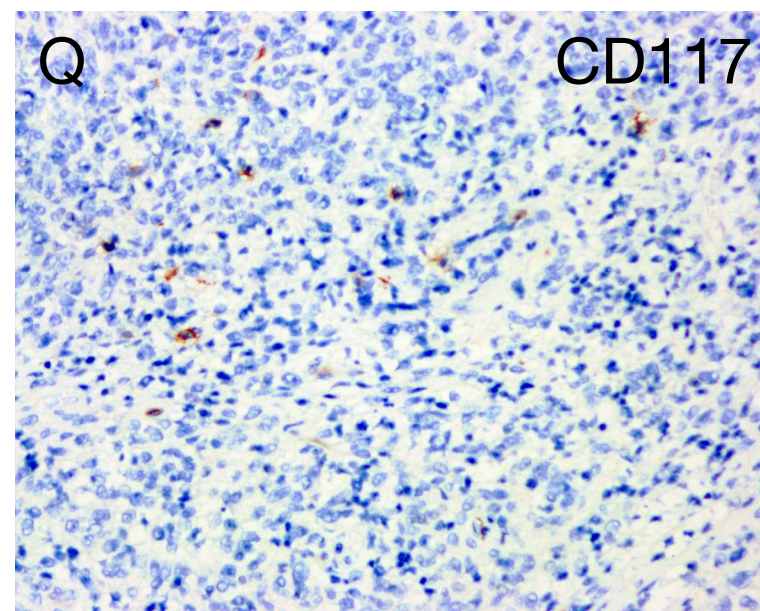

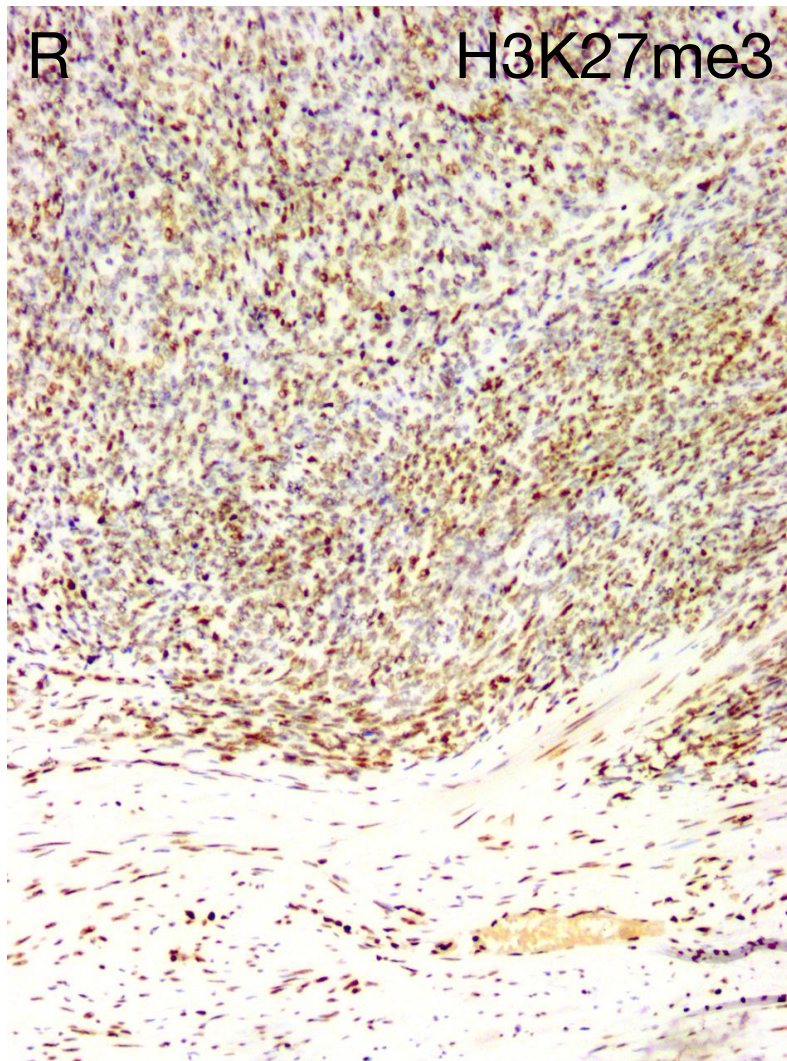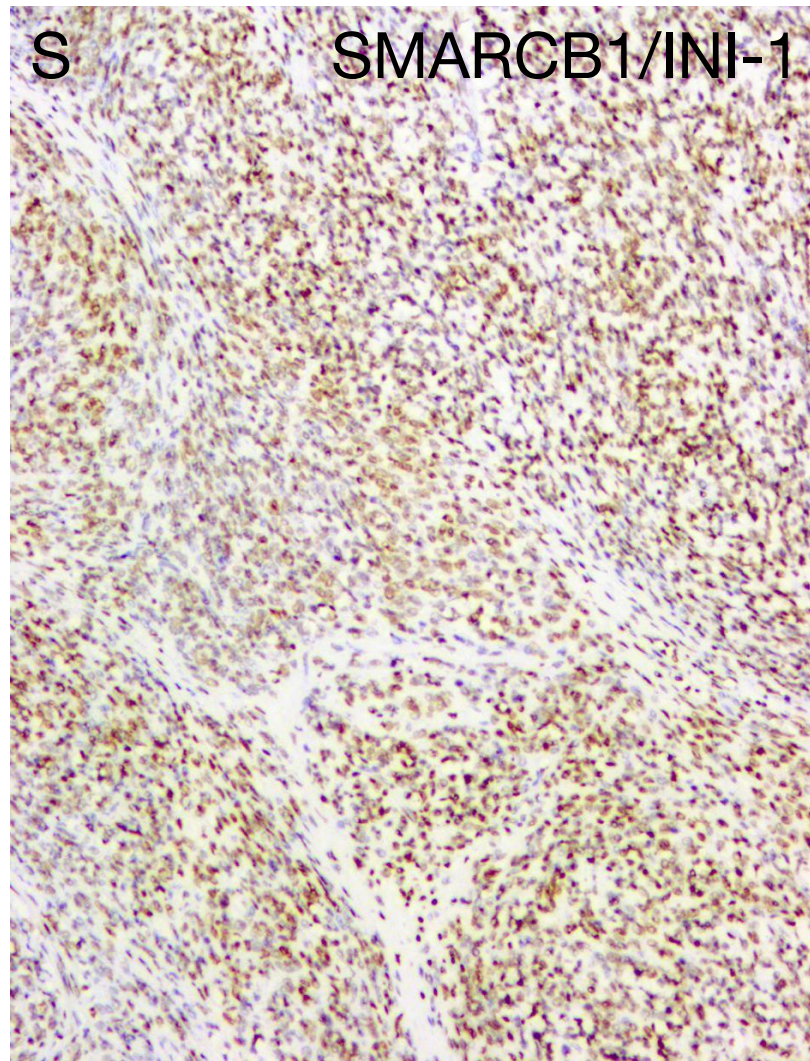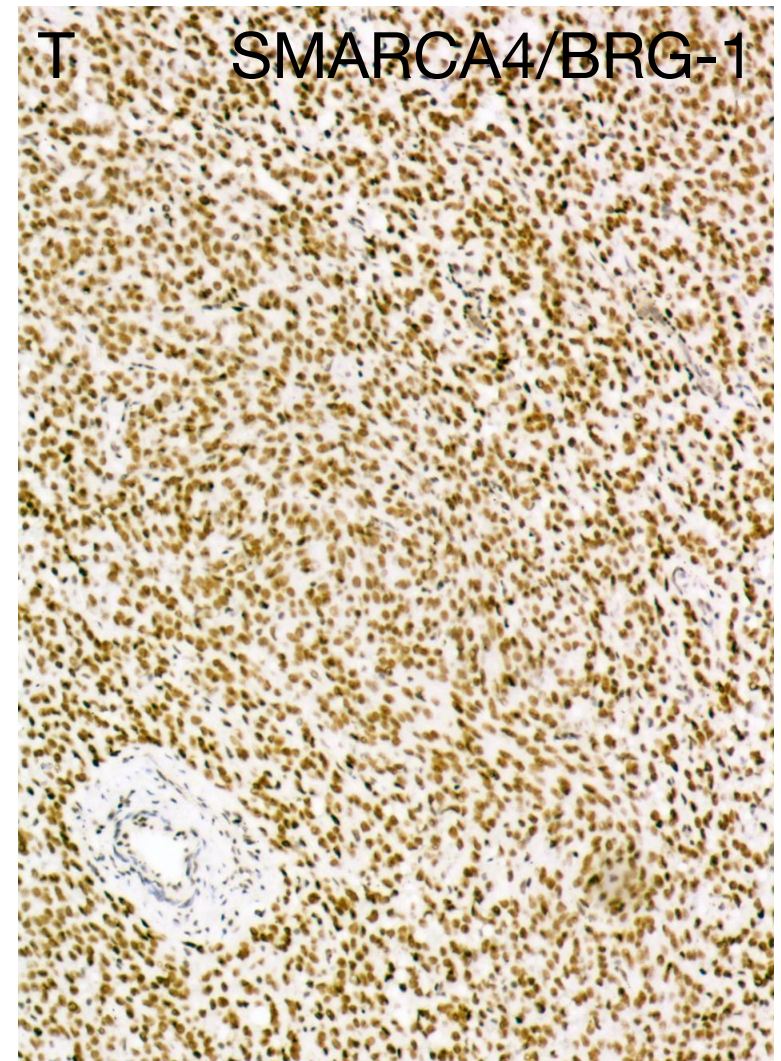

Supplement: Supplementary file 1 — Additional file 1. Additional pertinent immunohistochemical features of ARID1A deficient undifferentiated spindle cell and rhabdoid sarcoma of the prostate. The tumor cells are diffusely positive for (A) vimentin and focally positive for (B) epithelial membrane antigen, (C) Cam5.2, and (D) SATB2 (all in less than 10% tumor cells). (E) The Ki67 proliferation index was estimated at 50%. Immunohistochemical stains are negative in the tumor cells including (F) SOX10, (G) S100 protein, (H) P63, (I) cytokeratin5/6, (J) NKX3.1, (K) alpha-methylacyl-CoA racemase (AMACR), (L) smooth muscle actin, (M) desmin, (N) myogenin, (O) DOG1, (P) STAT6, and (Q) CD117. The expression of (R) H3K27me3, (S) SMARCB1/INI-1, and (T) SMARCA4/BRG-1is retained. [file 13000_2022_1198_MOESM1_ESM.pdf]
